# Investigating the eye in Down syndrome as a window to Alzheimer’s disease: the REVEAL protocol – a clinical cross-sectional study

**DOI:** 10.1136/bmjopen-2024-098285

**Published:** 2025-07-05

**Authors:** Aoife Mary Louise Hunter, Sarah Atkinson, Elaine Murray, Kathryn Saunders, Tunde Peto, Lajos Csincsik, Jamie Mitchell, Henrik Zetterberg, André Strydom, Julie-Anne Little, Imre Lengyel

**Affiliations:** 1 Centre for Optometry and Vision Science, Biomedical Sciences Research Institute, Ulster University, Coleraine, UK; 2 Centre for Genomic Medicine, Biomedical Sciences Research Institute, Ulster University, Coleraine, UK; 3 Personalised Medical Centre, School of Medicine, Ulster University, Coleraine, UK; 4 Centre for Public Health, School of Medicine Dentistry and Biomedical Science, Queen’s University Belfast, Belfast, UK; 5 The Wellcome-Wolfson Institute for Experimental Medicine, School of Medicine Dentistry and Biomedical Science, Queen’s University Belfast, Belfast, UK; 6 UCL Queen Square Institute of Neurology and UK Dementia Research Institute, University College London, London, UK; 7 Hong Kong Center for Neurodegenerative Diseases, Hong Kong, UK; 8 Wisconsin Alzheimer’s Disease Research Center, University of Wisconsin School of Medicine and Public Health, University of Wisconsin-Madison, Madison, Wisconsin, USA; 9 Department of Psychiatry and Neurochemistry, Institute of Neuroscience and Physiology, University of Gothenburg Sahlgrenska Academy, Gothenburg, Sweden; 10 Institute of Psychiatry, Psychology & Neuroscience, King’s College London, London, UK; 11 Wellcome-Wolfson Institute for Experimental Medicine, QUB, Belfast, UK

**Keywords:** Dementia, Aging, OPHTHALMOLOGY

## Abstract

**Abstract:**

**Introduction:**

There is a need for early, non-invasive and inexpensive biomarkers for Alzheimer’s disease (AD), which could serve as a proxy measure in prevention and intervention trials that might eventually be suitable for mass screening. People with Down syndrome (DS) are the largest patient group whose condition is associated with a genetically determined increased risk of AD. The REVEAL study aims to examine changes in the structure and function of the eye in individuals with DS compared with those with mild cognitive impairment (MCI) and cognitively healthy control (HC) individuals. REVEAL will also explore whether these changes are connected to inflammatory markers previously associated with AD.

**Methods and analysis:**

The protocol describes a cross-sectional, non-interventional, single-centre study recruiting three cohorts, including (1) participants with DS (target n=50; age range, 6–60 years), (2) participants with MCI (target n=50; age range, 50–80 years) and (3) HC participants (target n=50; age range, 50–80 years). The primary research objective is to profile retinal, choroidal and lenticular status using a variety of eye imaging modalities and retinal functional testing to determine potential associations with cognitive status. The REVEAL study will also measure and compare established blood markers for AD and proteomic and transcriptomic marker profiles between DS, MCI and HC groups. Between-group differences will be assessed with an independent sample t-test and χ^2^ tests for normally distributed or binary measures, respectively. Multivariate regression analysis will be used to analyse parameters across all three cohorts. Data collection began in October 2023 and is expected to end in October 2025.

**Ethics and dissemination:**

The study gained a favourable opinion from Health and Social Care Research Ethics Committee A (REC reference 22/NI/0158; approved on 2 December 2022; Amendment 22/0064 Amend 1, 5 April 2023; Amendment 22/0064 Amend 2; 23 May 2024; Amendment 22/0064 Amend 3; 25 June 2024; Amendment 22/0064 Amend 4; 16 January 2025; Amendment 22.0064 Amend 5; 9 May 2025; Amendment 22.0064 Amend 6; 9 June 2025). The study has also been reviewed and approved by the School of Biomedical Sciences Research Ethics Filter Committee within Ulster University. Findings from the REVEAL study will be presented to academic audiences at international conferences and peer-reviewed publications in targeted high-impact journals after data collection and analysis are complete. Dissemination activities will also include presentations at public events.

**Strengths and limitations of this study**

- The strength of the REVEAL study is that it will perform an in-depth comparison of ocular structure and function changes between Down syndrome (DS), mild cognitive impairment (MCI) and healthy control groups.
- Collection of saliva and tear biofluid samples, in addition to blood, will evaluate whether non-invasive, patient-friendly methods of biofluid sampling can be an effective alternative to measure Alzheimer’s disease (AD) status or changes in AD risk.
- A limitation to the work is that, while the REVEAL study will recruit participants with MCI who might not all ultimately develop AD, including a non-DS MCI group and older non-DS control group will be important to enable comparison across these three distinct study groups.

## Introduction

 Alzheimer’s disease (AD) is the most common form of dementia, with the prevalence predicted to double in Europe and triple worldwide by 2050.^1^ Early, non-invasive and inexpensive biomarkers of AD that are suitable for mass screening and could serve as a proxy measure in early prevention and intervention trials are much needed.^2^ There is growing evidence that ocular structures could serve as biomarkers.^3^

Reduced retinal nerve fibre layer (RNFL) and choroidal thickness have been found to be associated with brain atrophy in AD.^3 4^ However, the nature of the retinal and choroidal changes that occur before the clinical symptoms for AD arise is not well documented due to the scarcity of studies investigating the early stages of the disease in the eye.^3^,^5^ It has been suggested that retinal thickening due to inflammatory changes in mild cognitive impairment (MCI) and preclinical stages of AD may precede the thinning observed at later stages,^5^,^8^ but little information is available describing choroidal changes in MCI.^9^ A link between lens opacities (cataracts) and AD has also been reported.^10^,^13^ At present, only a few studies have investigated early retinal, choroidal and lenticular changes related to AD. Designing studies where presymptomatic AD could be investigated is problematic without knowing who will develop the disease. Moreover, large population-based studies with long follow-up periods are expensive.

Due to triplication of chromosome 21 resulting in overexpression of amyloid precursor protein and the build-up of amyloid-β in the brain, individuals with Down syndrome (DS) are predisposed to the development of AD.^14^,^16^ Fortea *et al*^17^ described the similarity between the natural history of AD in people with DS compared with those with sporadic or autosomal dominant AD, characterised by a long preclinical phase; and they highlight the unique opportunity for research of AD in DS to gain better insight into early changes associated with future AD. While conventional AD biomarkers have been studied in individuals with DS, limited work has explored potential ocular biomarkers in this patient population.^18^,^20^ Csincsik *et al*^18^ investigated choroidal changes in adults with DS (with no clinical symptoms of AD) and found a thicker retina and choroid in DS compared with age-matched controls. Although there are known developmental differences in the eye in DS,^21 22^ no significant difference in choroidal thickness was observed in work investigating spectral-domain optical coherence tomography (SD-OCT) findings in Asian Indian children with DS (aged 3–78 months) compared with controls (aged 4–82 months), yet the photoreceptor layer and complex were found to be thinner in children with DS compared with those without.^23^ A study conducted in Turkey found that, although central foveal retinal thickness was significantly thicker in people with DS aged 7–18 years compared with age-similar controls, no significant difference in central choroidal thickness was observed between DS and control groups.^24^ Little *et al*^19^ conducted an in-depth investigation of the crystalline lens in adults with DS and discovered the frequent presence of small ‘dot’ opacities in 54% of participants with DS, distinctly different from typical age-related cataracts. Considering such findings, lenticular opacities in addition to thicker retina and choroid observed in DS appear to be distinct from developmental differences and may be indicative of inflammatory processes connected to the development of AD.^25^

The REVEAL study aims to better understand the underlying pathology of lenticular, choroidal and retinal differences in individuals with DS by comparing the outcomes of a range of ocular assessments and body fluid biomarkers in this cohort to cognitively healthy older individuals and patients diagnosed with MCI. This work seeks to define the pathomechanism of AD in DS better and help to identify the early structural and molecular events occurring before AD becomes clinically manifest.

## Methods and analysis

### Study design

This is a cross-sectional, non-interventional, single-centre study in approximately 150 male and female participants, including those with DS aged 6–50 years old, those diagnosed with MCI aged 50–80 years old, and healthy control (HC) participants aged 50–80 years old without DS and evidence of MCI. The rationale for the younger age group of DS participants compared with the MCI and HC age-matched groups is that individuals with DS have a shorter life expectancy, and dementia occurs earlier in those with DS, with a cumulative incidence of 26% by the age of 50.^26^

#### Sample size

A sample size of 50 participants for the DS and MCI groups was derived from a power calculation using data on choroidal thickness differences from Csincsik *et al*^18^ and Little *et al.*^19^ Assuming that 80% power is required to detect a difference in choroidal thickness outcome between groups at a significance level of 0.05 and knowledge of previous imaging success rates of ~65% in DS populations, our calculations matched the estimated sample size needed for sufficient power.^27^

For the MCI and HC groups, we anticipate an estimate of Cohen’s d=0.70 based on a recent meta-analysis.^4^ Thus, if we set the statistical power at 0.8 and alpha at 0.05, according to a two-tailed hypothesis, we would need at least 35 participants in this group to obtain a Cohen’s d of 0.70, so targeting 50 would exceed this requirement.

#### Recruitment

DS participants will be recruited within the community (previously established links, charities and support groups), as well as through learning disability teams within Belfast and Northern Health and Social Care Trust (HSCT) areas. MCI participants will be recruited from memory clinics within the Belfast, Northern and Western HSCT areas in Northern Ireland, where they will have been diagnosed with MCI by consultant geriatricians and psychiatrists. HC participants will be recruited via social media channels and an email invitation to Ulster University and Queen’s University Belfast staff and students, in addition to local interest groups. All participants will be tested in the Northern Ireland Clinical Research Facility (NICRF). Data collection began in October 2023 and is expected to end in October 2025.

### Study objectives and outcome measures

The primary research objective of the REVEAL study is to profile retinal, choroidal and lenticular status using ocular imaging and functional testing and compare these with cognitive status. A suite of ocular assessments providing in-depth scans of the retina and lens of the eye will be conducted, along with visual electrophysiology to investigate structural and functional capacity. Cognitive assessments for DS participants will be performed using the Europe-wide Horizon 21 DS consortium protocol. This cognitive battery of assessments is based on tests demonstrated to be sensitive to cognitive changes in both DS and AD.^28^ Cognitive assessments will also be based on typically performed clinical assessments. All participants will undergo IQ assessments as a measure of general cognitive ability.

The secondary objective is to measure and compare established blood markers to assess AD status. Analysing plasma levels of AD biomarkers is increasingly being considered as a more accessible alternative to cerebrospinal fluid biomarker sampling to identify AD stage in humans^29^ and will provide a well-accepted standard to which phenotypic and molecular changes can be compared. Participant groups will be stratified based on these biomarkers.

Third, the REVEAL study also aims to measure and compare proteomic and transcriptomic marker profiles between DS, MCI and HC groups using saliva, tear and blood samples. It is recognised that blood sampling can be a barrier for those with DS to attend longitudinal studies and clinical trials. Considering this, identification of biomarkers in other less invasive biofluid samples would be beneficial.

### Study criteria

To determine eligibility for the REVEAL study, the following criteria have been applied:

#### General inclusion criteria

1. Male and female participants.
2. Able to provide consent or assent (where applicable) to participate in the study.

#### Specific inclusion criteria: DS group

1. Diagnosed with DS.
2. Aged 6–50 years.
3. All general inclusion criteria.

#### Specific inclusion criteria: MCI group

1. Diagnosed with MCI.
2. Aged 50–80 years.
3. All general inclusion criteria.

#### Specific inclusion criteria: HC group

1. Aged 50–80 years.
2. All general inclusion criteria.

#### General exclusion criteria

1. Previous bilateral cataract surgery.
2. Nystagmus.
3. Ocular albinism.
4. Diabetes.
5. Any other significant eye pathologies affecting imaging of the crystalline lens and retina.

#### Specific exclusion criteria: control group

1. History of AD, MCI.
2. Significant ocular disease that is, glaucoma, visual impairment due to age-related macular degeneration.

### Study procedures

**Figure 1:**
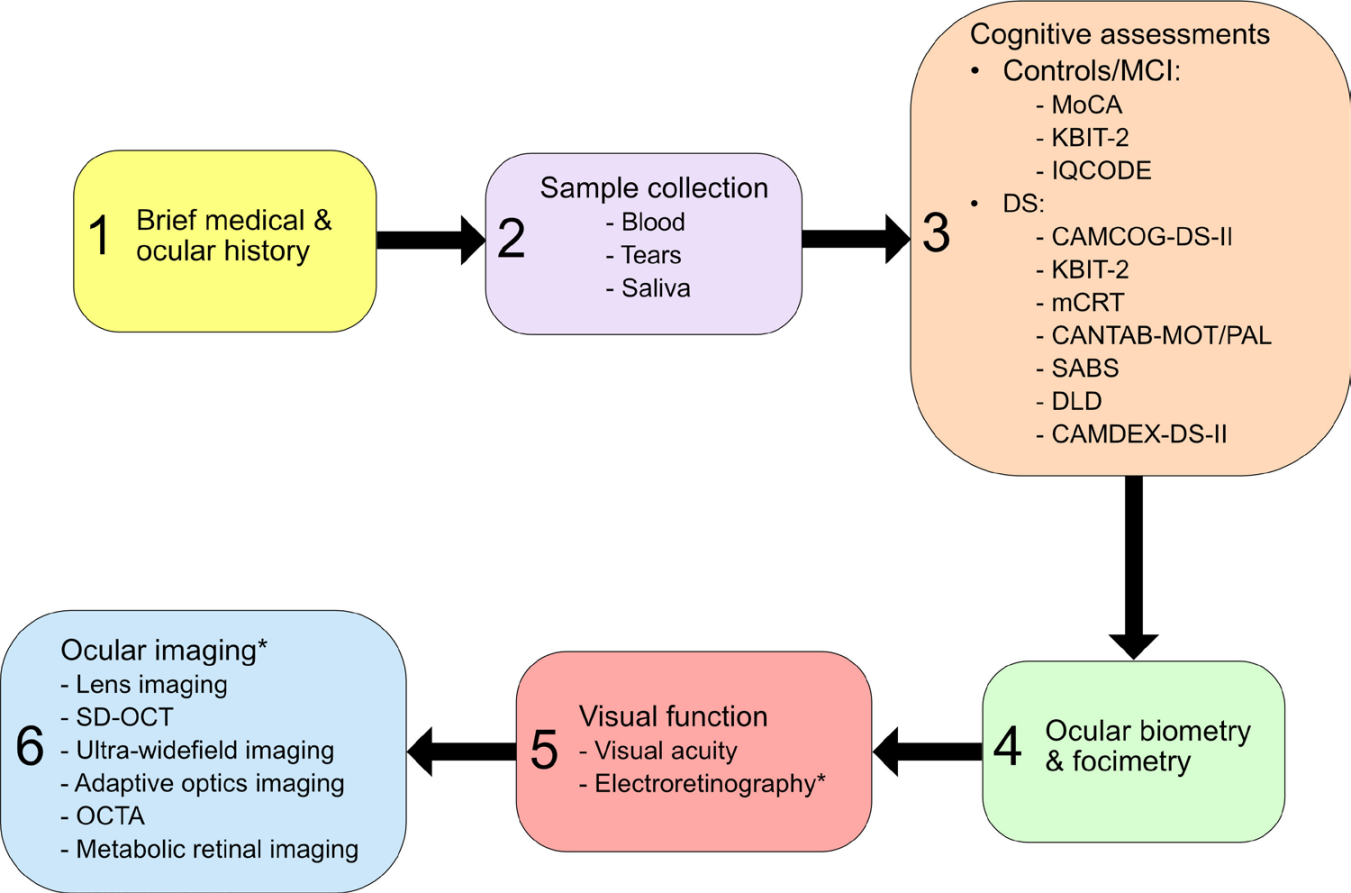
Schematic describing schedule of all study procedures. CAMCOG-DS-II, Cambridge Cognitive Examination adapted for individuals with DS–second edition; CAMDEX-DS-II, Comprehensive Assessment for Dementia in People with Down Syndrome and Others with Intellectual Disabilities–Second edition; CANTAB-MOT/PAL, Cambridge Neuropsychological Test Automated Battery-Motor screening task/Paired Associative Learning; DLD, Dementia Questionnaire for People with Learning Disabilities; DS, Down syndrome; IQCODE, Informant Questionnaire on Cognitive Decline in the Elderly; KBIT-2, Kaufman Brief Intelligence Test–Second Edition; MCI, mild cognitive impairment; mCRT, modified Cued Recall test; MoCA, Montreal Cognition Assessment; OCTA, OCT angiography; SD-OCT, spectral-domain optical coherence tomography; SABS, Short Adaptive Behaviour Scale. *Performed after pupil dilation with Tropicamide 1%.

A schedule of all study procedures is given in figure 1. Participants will attend the NICRF for one study visit lasting approximately 3 hours or two shorter visits if preferred. Initially, participants are interviewed to obtain a brief medical and ocular history and ensure the study eligibility criteria are met. A total of three biofluid samples will be collected between the hours of 9:30 and 11:30 to avoid diurnal shifts across participants and analysed as described below:

#### Blood collection

A small blood sample (x1 6 mL and x1 9 mL EDTA tubes) will be collected from participants by a trained phlebotomist and processed within 2 hours of collection. The 6 mL EDTA tube will be used to create plasma aliquots, by centrifuging the tube at 2000 × g for 10 min at 21°C followed by storage at −80°C. Buffy coat extraction will be performed by first centrifuging the 9 mL EDTA tube at 2000× g for 15 min at 4°C and discarding the plasma layer. The buffy coat layer will be washed by adding a 750 µL room temperature sterile phosphate-buffered saline (PBS) and inverting the tube 10 times. After centrifuging the samples at room temperature for 2 min at 2680 x g, the PBS will be removed, and the washing process repeated. After suspension in PBS, RNALater solution will be added to the buffy coat and mixed well before storage at −80°C.

Three approaches will be used for proteomic analysis of plasma samples to enable stratification of AD status including (1) analysis of plasma samples by the Biomarker Factory at the UK Dementia Research Institute at UCL Queen Square Institute of Neurology, using established AD markers including measurement of the plasma amyloid beta 42/40 ratio using immunoprecipitation mass spectrometry in addition to Single Molecule Array (Simoa, Quanterix, Billerica, Massachusetts, USA) measures of plasma P-tau217, neurofilament light and plasma glial fibrillar acidic protein as biomarkers for amyloid and tau pathology, neurodegeneration and astrocytic activation, (2) exploratory mass spectrometry analysis by Ulster University to identify intact proteins and peptides in order to characterise differences between the DS and non-DS control groups followed by a multiplex custom peptide for validation of such findings and (3) targeted proteomic analysis using Olink Explore HT panels (Olink Proteomics, Uppsala, Sweden), and NUcleic acid Linked Immuno-Sandwich Assay (NULISA, Alamar, Fremont, California, USA). The buffy coat samples will undergo RNAseq analysis for transcriptomic analysis.

#### Tear collection

Tear sampling will be performed on all participants using Schirmer strips without anaesthesia and before pupil dilation, in both eyes simultaneously, according to manufacturer instructions (Haag-Streit UK LTD, Harlow, Essex, UK). In summary, the rounded wick end of the Schirmer strips will be bent at the indentation at right angles and placed temporarily on the lower lid margin of both eyes, carefully avoiding the cornea, while the participant is seated and looking up. During tear collection, the participants will be permitted to continue normal blinking or to look down and close their eyes if preferred. Tear collection will continue for a maximum of 5 min or when the tear front reaches the end of the Schirmer strip. While the participant is looking up, the strips will be carefully removed with tweezers and processed according to the protocol recommended by Olink (Olink Proteomics, Uppsala, Sweden). In short, strips will be placed into Costar Spin-X centrifuge tubes (Corning, Corning, New York, USA). Elution buffer (300 µL of PBS mixed with 0.05% Tween-20 and 1% Bovine Serum Albumin) will be added to each tube with the Schirmer strip and placed in a tube shaker at room temperature for 10 min then centrifuged at 16 000 × g for 10 min at room temperature, before aliquots are created and frozen at −80°C. The tear samples will undergo untargeted proteomic and transcriptomic analysis using Olink Explore HT panels (Olink Proteomics, Uppsala, Sweden).

#### Saliva collection

Saliva samples (2 mL) will be collected (passive drool) according to the protocol recommended by Olink (Olink Proteomics, Uppsala, Sweden). In summary, saliva will be collected at least 1 hour after eating, drinking, chewing gum, using nicotine or dental hygiene products, and 5 min after oral rinsing with sterile water. Participants will be instructed to drool into a 15 mL Eppendorf Protein LoBind tube (Eppendorf SE, Hamburg, Germany) kept on ice before collection and reminded not to cough up mucus. A minimum of 2 mL of saliva will be collected, indicated by a line on the tube, but saliva will be collected for no more than 30 min. The samples will then be centrifuged at 2600× g for 15 min at 4°C. If incomplete separation occurs, the samples will be spun for an additional 20 min at 4°C. Roche cOmplete Mini Protease Inhibitor Cocktail made to a 1X final concentration will be added and mixed well with the saliva samples before aliquots are created and frozen at −80°C. Samples will be processed and frozen within 30 min from the end of collection. The saliva samples will undergo untargeted proteomic analysis using Olink Explore HT panels (Olink Proteomics, Uppsala, Sweden).

#### Cognitive assessments

For the DS group, cognition will be measured using the Horizon 21 DS consortium protocol,^28^ consisting of the following assessments performed with participants:

1. The Cambridge Cognitive Examination adapted for individuals with DS–Second edition (CAMCOG-DS-II): This latest version of the CAMCOG will enable objective assessment of the areas of cognitive function known to decline in dementia, beyond cognitive impairments due to intellectual disability.^30^ Orientation, language (comprehension and expression), memory (new learning), praxis (drawing/copying and actions to command), perception and executive function (fluency, attention, abstract thinking, prospective memory and inhibition) are measured. Each category of cognitive function will be scored and an overall score calculated.
2. The modified Cued Recall Test: All DS participants performing this test will undergo a learning and a testing phase. During the learning phase, 12 items representing distinct semantic categories are presented on 3 four-item cards, with each item accompanied by a unique category cue.^31^ Learning is repeated up to a maximum of three times if necessary to recall all four items. The testing phase consists of three trials of free and cued immediate recall, generating two measures, a free immediate recall score (FIRS; spontaneous recall of the list of 12 items for each trial) and a total immediate score (FIRS plus items recalled when the category cue was provided). A 20 min delayed recall trial is also included, generating two additional scores: free delayed recall score (FDRS) and a total delayed score (FDRS plus items recalled after the category cue was provided). The total number of intrusion errors (responses not corresponding to any of the items of the test) during both free and cued recall will be recorded.
3. The Cambridge Neuropsychological Test Automated Battery (CANTAB, 2016) motor screening task (CANTAB-MOT) and Paired Associative Learning (CANTAB-PAL): After completing the CANTAB-MOT screening test to orient all DS participants to the touch screen on the iPad, they will complete the CANTAB-PAL task. This is a measure of visuospatial short-term memory where participants are required to remember the locations of an increasing number of patterns in progressive stages, hidden behind boxes on the screen. The test terminates if a particular stage is not completed within a maximum of 10 attempts. The primary outcome from this test is the first trial memory score (number of pattern locations correctly remembered on the first trial for each stage attempted). The secondary outcome is the number of stages completed.

With the DS participant’s parent or carer, the Short Adaptive Behaviour Scale (SABS) will provide a functional cognitive measure. SABS^32^ is an informant questionnaire that asks carers to rate the participants’ adaptive behaviour abilities. There are two types of questions: (1) some ask informants to circle the highest-level ability that the individual has and (2) other questions ask informants to answer whether the individual can accomplish the task. There are a total of 24 questions and scores fit into three subdomains including personal self-sufficiency, community self-sufficiency and personal-social responsibility. Total scores for all domains will also be calculated.

Informants will complete two behavioural measures of cognitive function for the person they care for, including the Dementia Questionnaire for People with Learning Disabilities (DLD) and the Comprehensive Assessment for Dementia in People with Down Syndrome and Others with Intellectual Disabilities–Second edition (CAMDEX-DS-II). The DLD, formerly known as DMR, is a screening tool that measures specific cognitive deterioration as a result of dementia,^33^ as well as functional decline due to dementia and severe sensory or psychiatric problems. The DLD is an informant-based questionnaire, consisting of 50 items forming two domains with eight subscales including (1) cognitive (short-term memory, long-term memory, spatial and temporal orientation) and (2) social (speech, practical skills, mood, activity and interest, behavioural disturbance). The Sum of Cognitive Scores and the Sum of Social Scores will be calculated for each participant.

The CAMDEX-DS-II is an updated version of the CAMDEX interview scale, adapted for DS.^34^ It involves a structured informant interview and is comprised of six parts including (1) participant’s best level of functioning (education and employment, basic skills and independent living), (2) cognitive and functional decline (everyday skills, memory, orientation, general mental functioning, language, perception, praxis, executive functions, personality, behaviour and self-care), (3) current mental health (depression, anxiety, psychosis, clouding/delirium, obsessive–compulsive disorder, alcohol or substance abuse), (4) current physical health (physical disability, hypothyroidism, cerebrovascular problems, adverse effects of medication), (5) current neurological health (sleep-wake patterns, seizures) and (6) significant life events.

General verbal and non-verbal cognitive abilities will be measured across all participant groups using the Kaufman Brief Intelligence Test–Second Edition.^35^ The Montreal Cognition Assessment^36^ is a commonly used validated screening tool for cognitive decline and will be completed by all participants in the MCI and HC groups. Informants for participants in these cohorts will also complete the short form of the Informant Questionnaire on Cognitive Decline in the Elderly,^37^ routinely used in HSCT memory clinics.

#### Visual function measures

To assess macular function, monocular visual acuity will be measured according to the ETDRS protocol with spectacles if worn. Spectacle lens power will be measured using focimetry.

Retinal function will be examined objectively using visual electrophysiology (MonCVONE, Metrovision, Pérenchies, France) following pupil dilation using Tropicamide Hydrochloride 1%. The abbreviated version of the ISCEV Standard ERG protocol will be performed due to the inclusion of young children and to reduce test time and fatigue. This includes the following conditions:

1. Light adapted (LA) 3 ERG.
2. LA 30Hz ERG.
3. Dark-adapted (DA) 0.01 ERG following 10 min dark adaptation.
4. DA 10 ERG.

#### Ocular imaging

Previous work has revealed thickening of both the retina and the choroid on OCT images^18 20^ and atypical lenticular opacities with high-resolution slit lamp imaging^19^ in DS. Considering this, SD-OCT (Spectralis, Heidelberg Engineering, Heidelberg, Germany) and crystalline lens imaging (Zoom-Photo Slit lamp FS-3, Nikon, Tokyo, Japan) will be performed in all participants where possible. Optic nerve centred-OCT will also be used to measure peripapillary RNFL thickness as this may also be a useful diagnostic biomarker of AD.^4^ Changes in parameters of OCT Angiography, including the deep capillary plexus, foveal avascular zone and superficial capillary plexus, have been observed in individuals with AD and MCI compared with HC individuals^4^ and will be investigated in the current study. Retinal phenotypes, including hard and soft drusen, reticular pseudo-drusen, atrophy and vascular changes in the macula and the periphery, previously associated with AD,^38 39^ will be analysed on autofluorescence and colour ultra-widefield retinal images (California P200DTx, Optos, Dunfermline, Scotland). Recent work has observed reduced cone photoreceptor outer segment density in patients with multiple sclerosis (probably associated with inflammatory changes in the outer retina) using adaptive optics imaging (rtx1, Imagine Eyes, Orsay, Frank).^40^ To our knowledge, no previous study has examined photoreceptor density using adaptive optics imaging in AD and DS cohorts, and due to the possibility of early inflammatory changes in AD, we will investigate cone changes in this study. Given that metabolic dysfunction is associated with AD^41^ and DS,^42^ metabolic retinal imaging will also be undertaken by measuring oxygen saturation in retinal vessels (Oxymap T1 Oximeter, Oxymap, Reykjavík, Iceland). All ocular imaging will be undertaken after pupil dilation with 1% tropicamide.

### Data analysis plan

Our initial data analysis plan is described below, with flexibility to employ other approaches if needed as data collection progresses. In recognition of the fact that not all participants may complete the entire study protocol, the sample size calculation has accounted for this. Any cases where data is missing at random will be omitted, and the remaining data analysed. This approach for handling random missing data is known to produce unbiased estimates and conservative results.^43^

All anonymised images will be transferred to the Central Administrative Research Facility at Queen’s University Belfast for detailed image grading by the Belfast Ophthalmic Reading Centre. Regression analysis will be performed on visual function and imaging data. The general estimation equation (GEE) will be used to treat the eye as the unit of analysis and appropriately account for the correlation between the two eyes where data from both eyes of a participant is available.^44 45^ Between-group differences for individual variables will be assessed with an independent sample t-test and χ^2^ tests for normally distributed or binary measures, respectively. Where multiple tests of the same hypothesis are performed in each participant, post hoc Holm-Bonferroni correction will be applied to account for this and to control for independence. Potential confounders will be selected for regression analysis (GEE) if their association with exposure and principal outcome generates a p-value below 0.1. Multivariate regression analysis will investigate differences in lens opacities and retinal characteristics across all three cohorts and all comparisons with cognitive performance, where p<0.05 will indicate statistical significance.

χ^2^ tests will be performed to identify differentially expressed proteins between DS, MCI and HC groups. Classification accuracy will be evaluated using the receiver operating characteristic (ROC) curve and summarised by the area under the ROC curve (AUC) and the sensitivity corresponding to a specificity of 0.95 (i.e. ROC (0.05)). The ROC curve will be estimated using non-parametric methods. CIs for AUC and ROC (0.05) will be calculated using the parametric binormal models. For variables where multiple data points are available per participant, all data will be used, and methods proposed by Obuchowski^46^ will be performed to account for clustering of data within individual participants. Multivariate analysis will be employed to take into account confounding factors. Differences in continuous variables, such as clinical, protein and gene markers, between the risk and control groups will be assessed using an unpaired non-parametric t-test. Regression analysis will be used to determine the relationship between variables. Differences within a cohort will be analysed using a paired t-test. Where appropriate, two-sided tests will be used, with a significance threshold set at p<0.05. Analysis of continuous variables as mean (SD) will be compared using the unpaired two-sided Student’s t-test, or as median (IQR) and compared with Mann-Whitney U test where the data obviously deviate from a normal distribution. Categorical variables will be expressed as numbers (%) and compared by χ^2^ tests or Fisher’s exact test between moderate and severe case groups. Where a two-sided α of less than 0.05 will be considered statistically significant. Statistical analyses will be performed using R (R Core Team, 2020) and/or SPSS (V.26.0, IBM).

Bioinformatic analyses will be used to compare clinical and proteomic data sets and will be performed using Olink Analyse and R Studio to normalise data, generate volcano plots, perform principal component analyses and extract protein features or biomarker ‘signatures’ of immunopathology and protective immunity that have statistically significant differences between the DS, MCI and control groups. Association analyses using biological and clinical features will be performed using unsupervised clustering methods and logistic regression modelling.

## Ethics and dissemination

### Data management and ethical considerations

The research protocol adheres to the tenets of the Declaration of Helsinki, and each participant will provide written informed consent prior to data collection. For children with DS, written informed consent will be obtained from their parents or guardians, and the child will either provide written consent or assent depending on The Mental Capacity Act (2016) NI. For adults with DS, written informed consent will be taken unless the carers and research team determine that the individual does not have the capacity to provide consent for themselves. In such instances, written assent will be obtained from the person with DS and written consultee declaration obtained from parents or guardians. Participants, parents or guardians may withdraw from the study at any time.

All participant data will be anonymised by the assignment of a personal code. Hard copies of identifying data will be stored in a locked filing cabinet located within a secure university office. All electronic data will be stored on a password-protected university computer and backed up.

The study gained a favourable opinion from Health and Social Care Research Ethics Committee A (REC reference 22/NI/0158; approved on 2 December 2022; Amendment 22/0064 Amend 1, 5 April 2023; Amendment 22/0064 Amend 2; 23 May 2024; Amendment 22/0064 Amend 3; 25 June 2024; Amendment 22/0064 Amend 4; 16 January 2025; Amendment 22.0064 Amend 5; 9 May 2025; Amendment 22.0064 Amend 6; 9 June 2025). The study has also been reviewed and approved by the School of Biomedical Sciences Research Ethics Filter Committee within Ulster University.

### Dissemination

Findings from the REVEAL study will be presented to academic audiences at international conferences and peer-reviewed publications in targeted high-impact journals after data collection and analysis is complete. Dissemination activities will also include presentations at public events.

### Patient and public engagement

We have engaged and will continue to engage with Alzheimer’s Society, Public Involvement Enhancing Research, DS support groups, carers of a person with DS, the University of the Third Age and the College of Optometrists to gather ideas for ensuring patient and public involvement in the project from the outset. The College of Optometrists recently led a National Institute for Health and Care Research (NIHR)-funded study on eye care for those with dementia and has patient networks and guidance on what works for those with early and more advanced dementia.^47^ A project steering group with patient, carer and professional representation has been established to ensure there is oversight, collaboration and responsibility for the management of the project. Furthermore, we are establishing a public advisory group for feedback from participants on study procedures and advice on the dissemination of study findings.^48^
