# Supplementary material for: Investigating the eye in Down syndrome as a window to Alzheimer’s disease: the REVEAL protocol – a clinical cross-sectional study
Source: BMJ Open. 2025 Jul 5;15(7):e098285. doi: 10.1136/bmjopen-2024-098285 (PMC12228435; doi:10.1136/bmjopen-2024-098285)
Supplement: Supplementary file 1 [file bmjopen-2024-098285.reviewer_comments.pdf]

PEER REVIEW HISTORY

BMJ Open publishes all reviews undertaken for accepted manuscripts. Reviewers are asked to complete a checklist review form and are provided with free text boxes to elaborate on their assessment. These free text comments are reproduced below.

ARTICLE DETAILS

Title (Provisional)

Investigating the Eye in Down syndrome as a Window to Alzheimer's Disease: The REVEAL Protocol - A Clinical Cross-sectional Study

Authors

Hunter, Aoife Mary Louise; Atkinson, Sarah; Murray, Elaine; Saunders, Kathryn; Peto, Tunde; Csincsik, Lajos; Mitchell, Jamie; Zetterberg, Henrik; Strydom, André; Little, Julie-Anne; Lengyel, Imre

---

VERSION 1 - REVIEW

**Reviewer**	**1**
**Name**	**Saari, Toni**
**Affiliation**	**University of Helsinki Institute for Molecular Medicine Finland**
**Date**	**13-Jan-2025**
**COI**	**Since 2023, I have been involved in a research-to-business project aiming to use eye imaging for early detection of Alzheimer's disease. This project is still in very early stages and I do not think it biases my review, especially as the paper under review is a study protocol and by definition has no results.**

Hunter and colleagues present a thorough and mainly clear study protocol of the exciting, truly cross-disciplinary REVEAL study. The rich measurements are described in detail. However, some key pieces of information are still missing.

The start and expected end date of data collection should be clearly written in the main text on the paragraph concerning recruitment and in the abstract. Judging by the dates of approvals and the acknowledgement, I assume the study is already ongoing.

Definitions of MCI and HC are unclear, yet they are critical for the distinction between these two groups. Is the cognitive status based on a test or test(s) used in this study, or is it independent of these tests? Are some specific diagnostic criteria used for MCI, if so, which? Who makes the diagnosis and based on what information? Please clarify.

The authors write that "For the MCI and HC groups, we anticipate a conservative estimate of Cohen's d = 0.70 based on a recent meta-analysis.". Please elaborate on this: which variable of the meta-analysis was this based on, and was it based on MCI vs HC or HC vs AD? Changes that are evident in HC vs AD become trickier to observe in HC vs MCI due to the (even more) heterogenous nature of MCI and overlap with HC. Cohen's d of 0.7 in a non-cognitive outcome does not sound overly conservative when comparing healthy controls to MCI.

In Study objectives, the authors write: "Analysing plasma levels of AD biomarkers is currently the gold standard to identify AD in humans". This is inaccurate; CSF and PET are still the gold standards, but p-tau217 has started to be accepted as a more feasible alternative. Whether it functions like CSF in under 50-year-olds I believe is an open question. There seems to be a slight divergence between CSF and plasma p-tau217 in the upper end of age groups (Hazan et al., JNNP 2024; https://jnnp.bmj.com/content/early/2024/12/17/jnnp-2024-334122?rss=1). While the future looks promising for p-tau217, I would make that sentence more conservative at this point.

Data analysis plan states that Shapiro-Wilk and Kolmogorov-Smirnov tests will be used to test whether data "obviously deviate from a normal distribution". I would suggest removing this part as these tests tend to be of limited practical use in many statistical applications, often underpowered in small samples and overly cautious in large samples (i.e. implying a deviation when the data are reasonably normally distributed), and many of the tests the authors mention are robust to deviations from normality in large enough samples. So, in a sense, I think mentioning these distribution tests is an unnecessary pair of statistical handcuffs for the authors.

In the abstract and throughout the main text, the authors speak of three cohorts. Would it make more sense to think of the REVEAL study as one cohort with three diagnostic groups?

Finally, I have a few comments on the data analysis plan that may go beyond what is needed for the protocol, and the authors may disregard these comments at this stage if they so please. However, these are questions that will come up at some point of the REVEAL study. The authors write: "Between-group differences will be assessed with an independent sample t-test and chi-squared tests for normally distributed or binary measures respectively.". For variables where multiple data points are available per person, how will the authors choose which data to use in these t-tests and chi-squared tests? If all data is used, the clustering of data within persons should be taken into account. If only a part of the data is used, it should be clear how it is decided. The above goes for ROC methods as well (see e.g. Obuchowski, Biometrics 1997 doi: 10.2307/2533958 for rationale and Tabata et al., Diagnostic Pathology 2019 doi: 10.1186/s13000-019-0839-8 for application using R).

Reviewer **2**

Name **Prajjwal , Priyadarshi**

Affiliation **Bharati Vidyapeeth (Deemed to be University)**

Date 18-May-2025

COI None

Please consider specifying the anticipated recruitment and completion timeline in the "Methods" or "Ethics and Dissemination" section for transparency and reproducibility. While it is noted that not all participants may complete the entire protocol, please briefly describe how missing data will be handled in the statistical analysis.

VERSION 1 - AUTHOR RESPONSE

Reviewer 1: Dr. Toni Saari, University of Helsinki Institute for Molecular Medicine Finland

Hunter and colleagues present a thorough and mainly clear study protocol of the exciting, truly cross-disciplinary REVEAL study. The rich measurements are described in detail. However, some key pieces of information are still missing.

We thank the reviewer for their comment about our thorough and exciting study protocol.

(1) The start and expected end date of data collection should be clearly written in the main text on the paragraph concerning recruitment and in the abstract. Judging by the dates of approvals and the acknowledgement, I assume the study is already ongoing.

We agree that the start and end dates of data collection should be included within the manuscript and have clearly added these to the abstract (lines 86 – 87) and the recruitment sub-section of the main text (lines 202 – 203).

(2) Definitions of MCI and HC are unclear, yet they are critical for the distinction between these two groups. Is the cognitive status based on a test or test(s) used in this study, or is it independent of these tests? Are some specific diagnostic criteria used for MCI, if so, which? Who makes the diagnosis and based on what information? Please clarify.

We agree that how we distinguish between our MCI and HC groups could be clarified. While we anticipate that the tests we use in the study will help differentiate cognitive status between the MCI and HC groups, we are recruiting participants with MCI who will have attended memory clinics and been diagnosed with MCI by consultant geriatricians and psychiatrists based on tests that vary across health and social trusts within Northern Ireland, but include cognitive testing and may include brain imaging. We have clarified how we distinguish between MCI and HC groups within the recruitment sub-section (lines 198 – 199).

(3) The authors write that "For the MCI and HC groups, we anticipate a conservative estimate of Cohen's d = 0.70 based on a recent meta-analysis.". Please elaborate on this: which variable of the meta-analysis was this based on, and was it based on MCI vs HC or HC vs AD? Changes that are evident in HC vs AD become trickier to observe in HC vs MCI due to the (even more) heterogenous nature of MCI and overlap with HC. Cohen's d of 0.7 in a non-cognitive outcome does not sound overly conservative when comparing healthy controls to MCI.

We thank the reviewer for this comment. The meta-analysis was based on MCI and AD grouped together vs HC. While we appreciate that changes evident in HC vs AD become trickier to observe in HC vs MCI, our work investigating ocular changes compared to differences in cognitive function and body fluid biomarkers between individuals with HC, MCI and DS is exploratory. Considering this, we have used a Cohen's d of 0.70 based on a recent meta-analysis reviewing the literature on ocular biomarkers of MCI and AD. We have removed 'conservative' from the text (line 189).

(4) In Study objectives, the authors write: "Analysing plasma levels of AD biomarkers is currently the gold standard to identify AD in humans". This is inaccurate; CSF and PET are still the gold standards, but p-tau217 has started to be accepted as a more feasible alternative. Whether it functions like CSF in under 50-year-olds I believe is an open question. There seems to be a slight divergence between CSF and plasma p-tau217 in the upper end of age groups (Hazan et al., JNNP 2024; https://protect.checkpoint.com/v2/r02/___https://jnnp.bmj.com/content/early/2024/12/17/jnnp-2024-334122?rss=1___.YzJlOnVsc3RlcnVuaXZlcnNpdHk6YzpvOjFkOTA3YTc3MjY2ZjE0ZjQ5ZmFkNmMyYjkzODQ2M2ZjOjc6ZWM3ZTpmODBkOTFiMmNkNzA0M2FmZTQ1MDUyY2Q1ODk2MmRhMmQxZGZlMmQ4OWIyM2VjNTU2YTE3ZDk3YmRlM2FiNDliOnQ6RjpO). While the future looks promising for p-tau217, I would make that sentence more conservative at this point.

We agree that this is a valuable point and have removed that plasma biomarkers of AD is gold standard to make this sentence more conservative (lines 217 – 218).

(5) Data analysis plan states that Shapiro-Wilk and Kolmogorov-Smirnov tests will be used to test whether data "obviously deviate from a normal distribution". I would suggest removing this part as these tests tend to be of limited practical use in many statistical applications, often underpowered in small samples and overly cautious in large samples (i.e. implying a deviation when the data are reasonably normally distributed), and many of the tests the authors mention are robust to deviations from normality in large enough samples. So, in a sense, I think mentioning these distribution tests is an unnecessary pair of statistical handcuffs for the authors.

We thank for the reviewer for this comment and agree that specifically stating that Shapiro-Wilk and Kolmogorov-Smirnov tests will be used to check for normality in the dataset is unnecessarily restricting, so have removed this from the data analysis plan (line 487 – 488).

(6) In the abstract and throughout the main text, the authors speak of three cohorts. Would it make more sense to think of the REVEAL study as one cohort with three diagnostic groups?

While we appreciate the reviewer's comment, the participants within the three different groups all have different characteristics with the DS group being younger than the HC and MCI groups as well as the HC group not having a diagnosis as such. Considering this, we would prefer to keep the wording as three cohorts within the REVEAL study.

(7) Finally, I have a few comments on the data analysis plan that may go beyond what is needed for the protocol, and the authors may disregard these comments at this stage if they so please. However, these are questions that will come up at some point of the REVEAL study. The authors write: "Between-group differences will be assessed with an independent sample t-test and chi-squared tests for normally distributed or binary measures respectively.". For variables where multiple data points are available per person, how will the authors choose which data to use in these t-tests and chi-squared tests? If all data is used, the clustering of data within persons should be taken into account. If only a part of the data is used, it should be clear how it is decided. The above goes for ROC methods as well (see e.g. Obuchowski, Biometrics 1997 doi: 10.2307/2533958 for rationale and Tabata et al., Diagnostic Pathology 2019 doi: 10.1186/s13000-019-0839-8 for application using R).

We thank the reviewer for the above comments on our data analysis plan as these are important to think about ahead of time. When assessing between-group differences with an independent sample t-test and chi-squared tests, this will be for variables where individual data points are available per person e.g. sub-foveal choroidal thickness. Where multiple tests of the same hypothesis are performed e.g. examining choroidal thickness at various locations in each individual participant, post hoc Holm-Bonferroni correction will be applied to account for this and to control for independence. This has been clarified within the main text (lines 464 – 466). When using visual function and imaging data from both eyes of a participant where

available, General Estimating Equation will be used to treat the eye as the unit of analysis and appropriately account for clustered data from both eyes (lines 460 – 462). For ROC methods, all data will be used where variables with multiple data points for each participant is available and methods proposed by Obuchowski[1] will be performed to account for clustering of data within individual participants. Appropriate text has been added to the data analysis plan (lines 477 – 479).

Reviewer 2: Dr. Priyadarshi Prajjwal, Bharati Vidyapeeth (Deemed to be University)

(1) Please consider specifying the anticipated recruitment and completion timeline in the "Methods" or "Ethics and Dissemination" section for transparency and reproducibility.

We agree that the start and end dates of data collection should be included within the manuscript and have clearly added these to the abstract (lines 86 – 87) and the recruitment section of the main text (lines 202 – 203).

(2) While it is noted that not all participants may complete the entire protocol, please briefly describe how missing data will be handled in the statistical analysis.

We thank the reviewer for raising this point and have added to the data analysis plan (lines 449 – 453). While it is anticipated that all participants may not complete the entire study protocol and the sample size has been calculated to account for this, any cases where data is missing at random will be simply omitted and the remaining data analysed. This method has been known to produce unbiased estimates and conservative results.[2]

VERSION 2 - REVIEW

**Reviewer**	**1**
**Name**	**Saari, Toni**
**Affiliation**	**University of Helsinki Institute for Molecular Medicine Finland**
**Date**	**22-Jun-2025**
**COI**	

The authors have addressed my concerns.
